# Advances in Second Near-Infrared Window Photothermal Agents and Photothermal Therapy for Tumors in Interdisciplinary Medical Research

**DOI:** 10.3390/pharmaceutics17091178

**Published:** 2025-09-10

**Authors:** Runxuan Zhou, Yufei Chen, Shuxi Yao, Weiyun Zhang, Dawei Ye

**Affiliations:** 1 Cancer Center, Tongji Hospital, Tongji Medical College, Huazhong University of Science and Technology, Wuhan 430000, China; m202376689@hust.edu.cn (R.Z.); yaoshuxi@hust.edu.cn (S.Y.); 2 Department of Pharmacy, Tongji Hospital, Tongji Medical College, Huazhong University of Science and Technology, Wuhan 430000, China; m202376494@hust.edu.cn; 3 Department of Laboratory Medicine, Tongji Hospital, Tongji Medical College, Huazhong University of Science and Technology, Wuhan 430000, China

**Keywords:** photothermal therapy, cancer therapy, photothermal conversion, NIR-II, clinical progress

## Abstract

Cancer continues to pose a significant threat to human health. While early diagnosis has improved survival rates for many cancer patients, a substantial number still do not achieve the desired treatment outcomes. Therefore, it is imperative to develop novel therapeutic approaches for tumor management. Second near-infrared window photothermal therapy has garnered considerable attention from researchers due to its effective tumor-killing capabilities and minimal side effects. This review commences by summarizing the advancements in second near-infrared photothermal agents, alongside an evaluation of the advantages and disadvantages of various photothermal agents. Subsequently, we highlight the benefits of combining photothermal therapy with other treatment modalities. Finally, we present a compilation of reports detailing the application of photothermal therapy in the treatment of various tumor types in clinical settings. In the conclusion, we underscore the challenges and potential research directions associated with photothermal therapy. Our article aims to facilitate interdisciplinary research in the fields of nanomedicine and clinical medicine.

## 1. Introduction

Cancer remains a significant threat to human health worldwide. Among 22 categories of diseases and injuries, cancer is the second leading cause of death and disability-adjusted life years [1]. In 2022, it was estimated that nearly 20 million new cases of cancer would be diagnosed globally, while 9.7 million people would succumb to the disease. It is projected that approximately one in five men and women will develop cancer in their lifetime, with about one in nine men and one in twelve women ultimately dying from it. Furthermore, the World Health Organization (WHO) predicts that the global incidence of cancer will rise to 35 million by 2050 [2]. Traditional combination therapies, including surgery, chemotherapy, and radiotherapy, are widely employed in the clinical treatment of tumors. However, these therapies encounter significant challenges, such as limited efficacy, drug resistance, and adverse side effects [3,4,5,6]. Therefore, the development of effective and non-invasive methods for cancer treatment is imperative.

Photothermal therapy (PTT) is a novel tumor treatment method that has evolved significantly over the past few decades. The fundamental principle of PTT involves the use of photothermal agents (PTAs) that convert near-infrared (NIR) laser light into localized hyperthermia within tumor tissues to induce cell death [7]. Due to its high efficiency, minimal invasiveness, reduced side effects, and enhanced tumor specificity, PTT has attracted widespread attention from researchers [8]. Initially, studies on PTT predominantly utilized lasers operating within the first near-infrared (NIR-I, 700–950 nm) window [9]. However, the maximum permissible exposure (MPE) for the commonly used 808 nm laser is limited to 0.33 W/cm^2^, as outlined by the American National Standards Institute (ANSI) Guidelines for the Safe Use of Lasers in Healthcare. This limitation poses challenges for the clinical application of PTT in cancer treatment [10]. Consequently, the second near-infrared (NIR-II, 1000–1350 nm) window has become increasingly preferred due to its advantages of deeper tissue penetration, reduced light scattering, and higher MPE (1.0 W/cm^2^), which may lead to improved therapeutic outcomes and decreased damage to surrounding healthy tissues [11]. In recent years, a growing number of PTAs have been reported for use in NIR-II PTT, demonstrating promising therapeutic efficacy [12,13,14].

**Figure 1:**
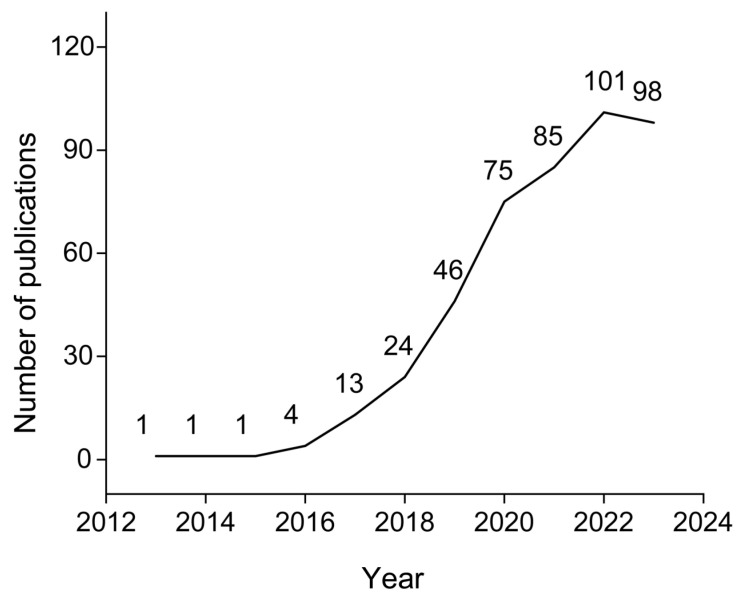
Annual number of publications on NIR-II window photothermal therapy.

**Figure 2:**
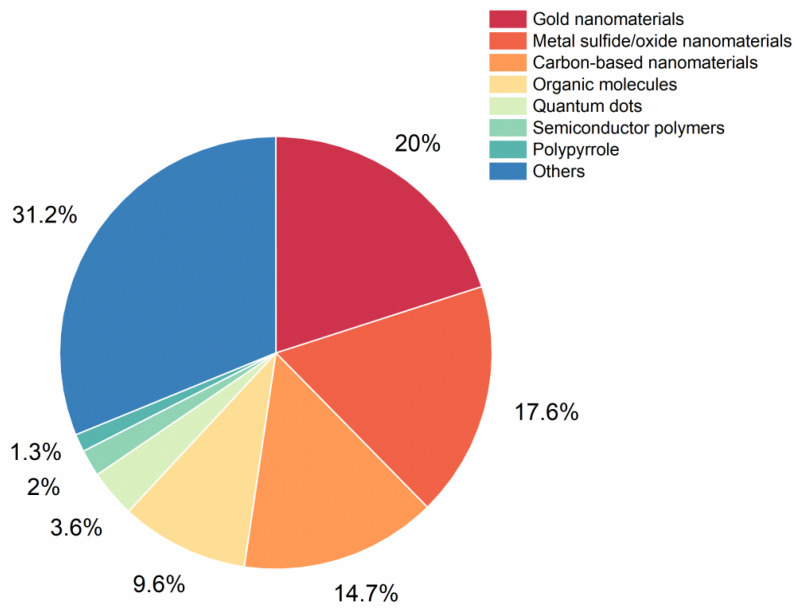
The number of publications on photothermal agents used in NIR-II window photothermal therapy from 2013 to 2023.

In this review, we summarize the synthesis of NIR-II PTAs, including metal nanomaterials, metal sulfide/oxide nanomaterials, carbon-based nanomaterials, quantum dot semiconductor polymers, and organic molecules (Figure 1 and Figure 2). We discuss advancements in photothermal therapy, emphasizing the synergistic effects of combining it with photodynamic therapy (PDT), chemotherapy, and immunotherapy. Furthermore, we review clinical application studies of PTT in various carcinomas, including skin cancer, prostate cancer, breast cancer, liver cancer, and lung cancer. The purpose of this review is to summarize recent and significant research achievements concerning NIR-II photothermal reagents, outline the properties of common photothermal agents, identify cancers suitable for clinical research, and provide reference research models for scholars in the field of nanomaterials. Additionally, we aim to present potential photothermal reagents for clinical researchers, thereby facilitating cross-disciplinary research between near-infrared photothermal reagents and clinical medicine.

## 2. NIR-II Photothermal Agents

PTAs play a crucial role in PTT. Most materials that absorb in the NIR-II window can convert NIR-II irradiation into heat with a specific efficiency constant known as photothermal conversion efficiency (PCE). However, ideal PTAs should exhibit a high PCE to induce hyperthermia capable of killing tumor cells within the permissible laser dose. Furthermore, photothermal stability, biocompatibility, ease of modification, and biodegradability within the body are significant considerations for PTAs [15]. In this section, we discuss the reported NIR-II PTAs categorized into the following groups: noble metal nanomaterials [16], metal sulfide/oxide nanomaterials [17], carbon-based nanomaterials [18], quantum dots [19], polymers [20], and organic molecules [21] (Table 1).

### 2.1. Inorganic Agents

Generally speaking, inorganic substances exhibit numerous desirable attributes, including enhanced photosensitivity, electrical conductivity, optical characteristics, magnetic properties, and thermal behavior. They serve not only as drug delivery systems but also as vehicles for therapeutic agents. Beyond these benefits, inorganic agents are readily synthesizable, possess a large surface area, and exhibit stable mechanical and chemical properties [22,23]. In cancer therapy, inorganic PTAs show significant advantages. PTAs generate heat locally through PTT [24], which can effectively target and kill tumor tissue and reduce damage to healthy tissue [25,26]. The relatively high absorption coefficient, stability, and longer circulation time of PTAs significantly enhance their therapeutic effect [26].

#### 2.1.1. Metal Nanomaterials

Gold nanoparticles (AuNPs) are the most commonly used PTAs in PTT due to their tunable surface plasmon resonance (SPR) peaks, good biocompatibility, and stability. Among various AuNPs architectures, gold nanorods are particularly prominent as their aspect ratio-dependent SPR peaks can be precisely adjusted across both the NIR-I and NIR-II windows. The pioneering work by the Masuda team, which first synthesized gold nanorods via electrochemical reduction, marked a breakthrough and ignited extensive exploration into shape-controlled synthesis of gold nanostructures [27]. Recent studies have further demonstrated that SPR properties can be deeply modulated through structural design. The Jia team fabricated various Au-on-AuNR hybrid nanostructures by precisely controlling interfacial energy and growth kinetics [28]. Notably, the branched wire-like “nanocoral” structures exhibited black-body-like broadband absorption, achieving a remarkable PCE of 67.2% under NIR-II window excitation (nanocorals 3c). This breakthrough provides a novel strategy for the full-spectrum tuning of plasmonic nanomaterials. Additionally, core–shell structural design has opened new avenues for performance optimization. Ji et al. achieved the first controlled synthesis of Au@Cu_2−x_S core–shell nanocrystals [29]. Experimental and theoretical studies have confirmed that these nanostructures exhibit both resonant and off-resonant SPR coupling effects in both the NIR-I and NIR-II regions. Even under excitation at a long wavelength of 1064 nm, their PCE reached 43.25%, demonstrating exceptional potential for cancer therapy. A novel plasmonic modulation strategy involving gold nanomaterials with MnO_2_ coating has been explored. He et al. demonstrated that the localized surface plasmon resonance (LSPR) of gold nanorods can be spectrally shifted from the NIR-I to the NIR-II window by controlling the thickness of the MnO_2_ shell [30]. The core–shell-structured GNR@SiO_2_@MnO_2_ (GSM) exhibited remarkable advantages: under NIR-II excitation, the hybrid material not only maintained excellent photothermal stability but also achieved a PCE of 27.47%.

**Figure 3:**
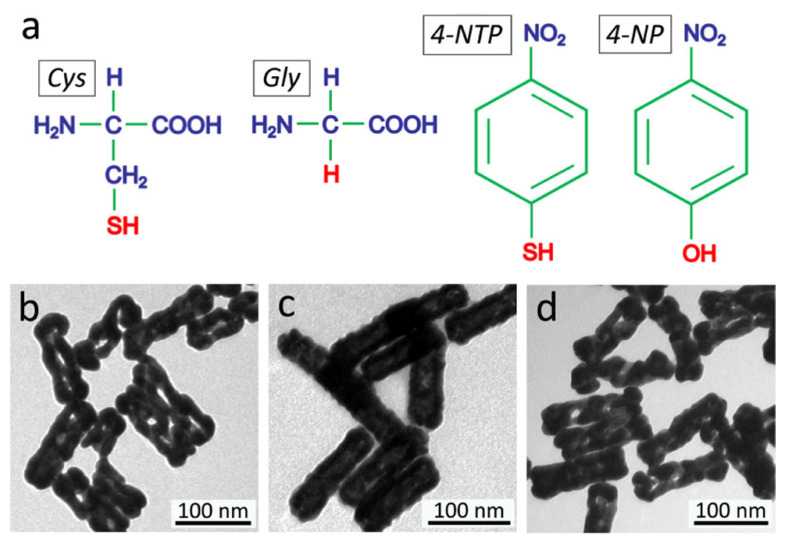
(**a**) Chemical structural formulas of l-cysteine (Cys), glycine (Gly), 4-nitrothiophenol (4-NTP), and 4-nitrophenol (4-NP). (**b**–**d**) TEM images of Au nanostructures obtained by using Gly (**b**), 4-NTP (**c**), and 4-NP (**d**) as the modification agents at the same concentration as Cys (2 μM) in the synthesis. Reprinted with permission from ref. [36]. Copyright 2025, American Chemical Society (Washington, DC, USA).

**Figure 4:**
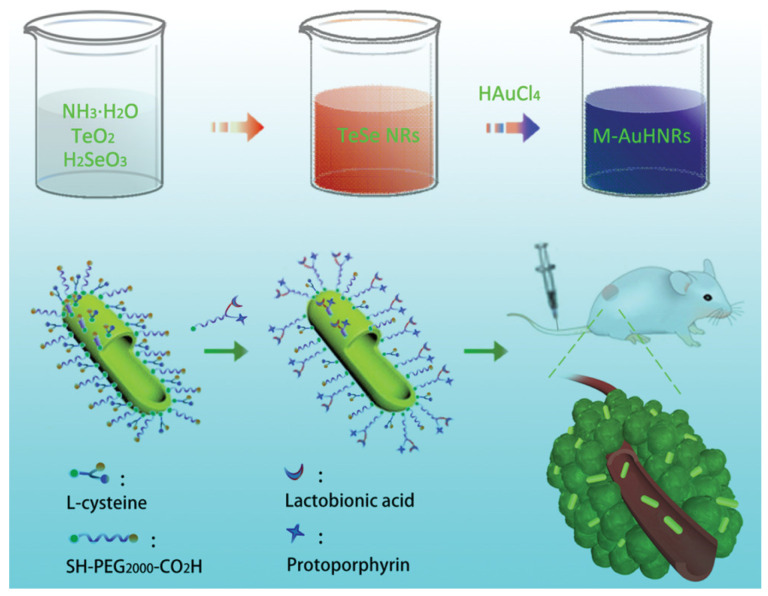
Schematic illustration of the preparation of miniature hollow gold nanorods (M-AuHNRs) and the functionalization with a tumor-targeted molecule and fluorescent molecule, as well as the application of M-AuHNRs in living tumor-bearing mice. Reprinted with permission from ref. [37]. Copyright 2018, Wiley-VCH GmbH (Weinheim, Germany).

In recent years, hollow gold nanostructures have emerged as a focal point of research due to their unique physicochemical properties. Compared to solid AuNPs of comparable size, hollow architectures not only exhibit a higher surface-area-to-volume ratio but also demonstrate significantly enhanced plasmonic characteristics through plasmon hybridization effects [31]. Although various configurations of AuNPs have been developed, most reported systems exhibit LSPR peaks confined to the first NIR-I window, with limited success in achieving responsiveness in the NIR-II window [32,33,34,35]. To address this challenge, Cai et al. innovatively synthesized aspect ratio-tunable hollow gold nanorods (AuHNRs) using a selenium (Se)-doped tellurium (Te) nanorod templating approach assisted by L-cysteine (Figure 3) [36]. Their study demonstrated that AuHNRs with an aspect ratio of 3 could achieve plasmonic resonance absorption in the NIR-II window, requiring only half the aspect ratio necessary for solid gold nanorods. These nanostructures delivered a 33% PCE, overcoming the optical limitations of conventional gold nanomaterials for deep-tissue therapeutic applications. Notably, no sub-50 nm hollow AuNPs with NIR-II responsiveness had been reported prior to this work. Building on their achievement, the team further developed microscale hollow gold nanorods (M-AuHNRs) using Te-Se nanorod templates [37]. With a mean length of 46.1 nm and an outer diameter of 24.7 nm, M-AuHNRs exhibited intensified plasmonic absorption at 1064 nm and an improved PCE of 34% (Figure 4). Through precise control of nanoscale dimensions and wall thickness, these structures synergistically optimized energy conversion efficiency and deep-tissue penetration capability while maintaining excellent biocompatibility, establishing a novel material design paradigm for tumor PTT. This study provides a plasmonic modulation strategy for metal nanomaterials in biomedical applications, particularly in the NIR-II window.

#### 2.1.2. Metal Sulfide/Oxide Nanomaterials

Due to their excellent free electron transfer properties and structural integrity [38,39], metal sulfides and oxide materials have garnered significant attention from researchers. Metal sulfide nanostructures are inorganic nanomaterials composed of one or more metal elements combined with sulfur. They encompass a diverse array of compounds, such as copper sulfide (CuS), silver sulfide (Ag_2_S) [40], lead sulfide (PbS), and iron sulfide (FeS). Lei et al. synthesized nickel sulfide (Ni_9_S_8_) NPs using a modified two-phase approach, achieving broadband absorption across the entire UV-Vis-NIR spectrum from 400 to 1100 nm [41]. The resultant Ni_9_S_8_ NPs exhibit a high extinction coefficient (22.18 L/g·cm) and a PCE of 46% at 1064 nm, indicating substantial potential for their application as effective photothermal agents responsive to the NIR-II window in biological contexts.

**Figure 5:**
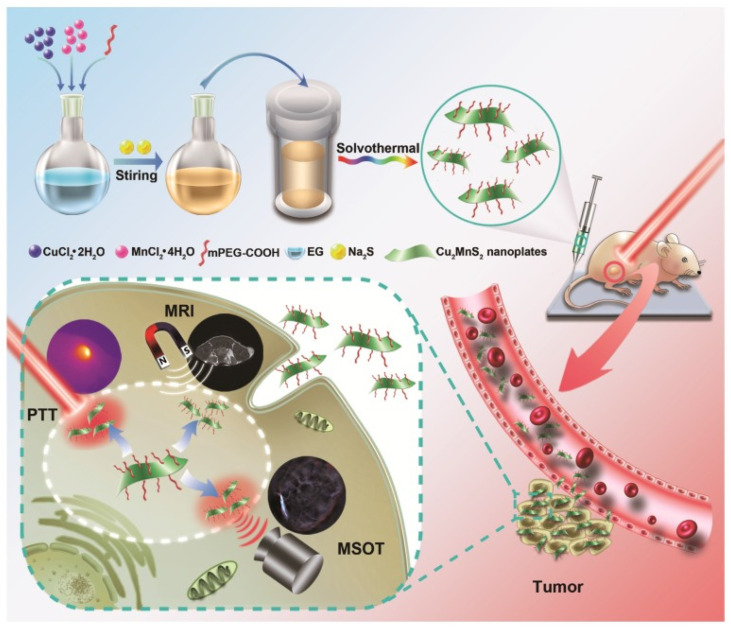
Schematic illustration for the syntheses and applications of Cu_2_MnS_2_ NPs as a theranostic platform. Reprinted with permission from ref. [42].

**Figure 6:**
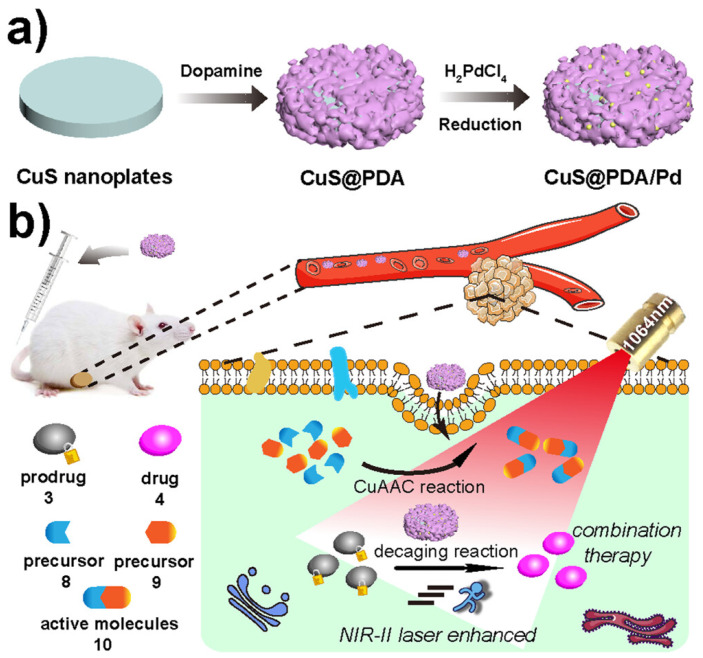
Construction of integrated CuS@PDA/Pd catalysts and in situ dual drug synthesis by multiple bioorthogonal transformations promoted by NIR-II Light: (**a**) schematic representation of the synthesis of CuS@PDA/Pd and (**b**) intracellular bioorthogonal CuS@PDA/Pd-catalyzed depropargylation accelerated by NIR-II light and the click reaction for in situ dual-drug synthesis. Reprinted (adapted) with permission from ref. [43]. Copyright 2022, American Chemical Society.

The unique vacancy structure of copper sulfide, along with the LSPR induced by the oscillation of conduction electrons, provides a promising avenue for studying PTT in NIR-II. Ke et al. synthesized Cu_2_MnS_2_ NPs using a simple and effective one-pot solvothermal method by adjusting the proportions of copper, manganese, and sulfur [42]. The resulting Cu_2_MnS_2_ NPs exhibit low cytotoxicity, a high PCE of 49.38%, excellent photostability, and strong absorbance in the NIR-II region. Furthermore, Cu_2_MnS_2_ NPs can effectively induce apoptosis in cancer cells both in vitro and in vivo under 1064 nm laser irradiation at a low power density of 0.6 W/cm^2^, indicating their significant potential for biomedical applications (Figure 5). Zhao et al. reported an NIR-II light-promoted integrated catalyst, CuS@PDA/Pd (where PDA refers to polydopamine), which achieved a PCE of 50.6% under 1064 nm laser irradiation at 1.0 W/cm^2^ (Figure 6a) [43]. In the treatment of orthotopic breast cancer, the integrated CuS@PDA/Pd nanocomposites enhance therapeutic efficacy by catalyzing the production of antitumor resveratrol analogs via the copper-catalyzed azide-alkyne cycloaddition (CuAAC) reaction, while simultaneously activating a prodrug of 5-fluorouracil (5FU) (Figure 6b). Currently, ternary bimetallic chalcogenide nanomaterials have been shown to significantly enhance the PCE compared to binary chalcogenides [44,45]. Xu et al. modified artificial nanozyme platinum nanoparticles on the surface of CuCo_2_S_4_ nanoparticles to prepare ultrasmall copper-based ternary bimetallic chalcogenides, specifically CuCo_2_S_4_-Pt-PEG nanocomposites [46]. These nanocomposites exhibit high mass extinction coefficients (7.69 L/g·cm) and outstanding PCE (η = 78.46%) when exposed to 1064 nm NIR-II light irradiation at a low power density of 0.8 W/cm^2^. Furthermore, the CuCo_2_S_4_-Pt-PEG nanocomposites demonstrated a prominent tumor suppression effect in vivo in 4T1 tumor-bearing mice upon 1064 nm laser irradiation. Recently, nanomedicines camouflaged with cell membranes have demonstrated intrinsic biocompatibility, prolonged circulation, immune evasion, and enhanced tumor accumulation [47]. Li et al. reported a tumor microenvironment responsive biomimetic nanoformulation of RCuS@tMCP, which was developed by conjugating the NIR-II photothermal agent copper sulfide nanoparticles (CuS NPs) as the “raspberry seeds” on the surface of red blood cell membrane (RBCm) camouflaged nanocomplex of CpG/protamine (denoted as tMCP) through matrix metalloproteinase-2 cleavable peptide [48]. The RCuS@tMCP exhibited excellent biocompatibility and photothermal conversion properties (η = 69.6%). Under laser irradiation (1064 nm, 0.8 W/cm^2^), RCuS@tMCP significantly suppressed the growth of both primary (90.1%) and distant (86.1%) 4T1 tumors while effectively preventing malignant metastasis (90%). Non-stoichiometric copper sulfide (NCS, Cu_2−x_S) is recognized for its exceptional LSPR absorption in the NIR region [49], particularly with an absorption peak in the NIR-II window, making it an ideal candidate for NIR-II PTAs. Consequently, Liu et al. developed biomineralized copper sulfide nanoparticles (BCS NPs) as NIR-II PTAs, capable of initiating NIR-II PTT with high tissue penetration depth [50]. Furthermore, the extinction coefficient (ε) and PCE of BCS NPs were measured at 16.6 L/g·cm and 29.8%, respectively. Under NIR-II irradiation (1 W/cm^2^, 5 min), BCS NPs reduced the viability of 4T1 breast cancer cells to below 50% and effectively eliminated breast cancer tumors within three days, demonstrating potent tumor ablation effects. He et al. reported a surface-enhanced Raman scattering (SERS)/NIR-II optical nanoprobe assembled from gold nanostars, Raman molecular tags, and silver sulfide quantum dots interconnected by silica bridges, named AuDAg_2_S [51]. The AuDAg_2_S exhibits a strong LSPR, achieving a satisfactory PCE of 67.1% at 1064 nm, which effectively induces apoptosis in CT26 colon cancer cells. Controlling the out-of-plane size effects of two-dimensional (2D) materials is anticipated to enhance their photothermal functionality in the NIR-II region. In this context, Su et al. prepared plasmonic atomic-thin (approximately 1.6 nm) 2D CuS nanocrystals (AT-CuS NCs) and discovered that these atomic-thin nanomaterials exhibit remarkable PCEs of up to 94.3% [52].

Metal oxides have garnered significant attention due to their photothermal properties. Recent studies have revealed the photothermal effect of Fe_3_O_4_ nanoparticles, highlighting their potential applicability in tumor treatments [53,54,55,56]. Under near-infrared laser irradiation, Fe_3_O_4_ magnetic nanoparticles generate sufficient energy to thermally ablate cancer cells. Wang et al. synthesized a novel NIR-II (1066 nm) responsive hollow magnetite nanocluster (HMNC) using a one-step solvothermal method, achieving high yields under an external magnetic field (0.5 T) [57]. Both in vitro and in vivo experiments demonstrated the excellent anti-tumor efficacy and a high PCE of 36.3% for HMNC. Zhou et al. reported an activatable NIR-II plasmonic theranostic system based on silica-encapsulated self-assembled gold nanochains (AuNCs@SiO_2_) for precise tumor diagnosis and effective treatment [58]. The synthesized nanomaterials possess two significant advantages: AuNCs@SiO_2_ exhibits TME activation properties that are inactive in normal tissues, thereby minimizing damage to healthy tissues. Furthermore, the self-assembled AuNCs@SiO_2_ nanostructure demonstrated superior NIR-II tissue penetration for PTT in tumor tissues, achieving a significant PCE of 82.2%, the highest reported among plasmonic phototheranostics. Yu et al. synthesized non-stoichiometric hollow silicon oxide nanoparticles (H-SiOx-PEG NPs) via a magnesiothermic reduction process [59]. These black nanoparticles exhibited a desirable LSPR in the NIR-II window, achieving a PCE of up to 48.6% at 1064 nm. H-SiOx-PEG NPs are highly efficient NIR-II PTAs suitable for in vivo cancer PTT, demonstrating effectiveness at a low power density (0.6 W/cm^2^) and a short irradiation time (5 min), sufficient for tumor ablation. This efficiency is the highest reported among noble metal and semiconductor-based NPs as NIR-II PTT PTAs.

#### 2.1.3. Carbon-Based Nanomaterials

Carbon-based nanomaterials, primarily consisting of carbon nanotubes and graphene, have garnered significant interest due to their low biotoxicity, affordable manufacturing costs, high PCE, and multifunctional surface modification properties. These attributes have facilitated their widespread application in cancer PTT [60,61]. The sp^2^ structural domain in nanocarbon materials effectively absorbs NIR light and excites surface plasmons, thereby converting the transmitted random dipole resonance into thermal energy, which is essential for achieving PTT in the near-infrared region. However, since most carbon nanomaterials are primarily sensitive to NIR-I light and are ineffective for treating deep tissues, researchers are focusing on synthesizing carbon-based nanomaterials with high PCE in the NIR-II region [62,63]. The efficacy of PTT for tumors is typically characterized by its ability to induce apoptosis in cancer cells in vitro and to reduce tumor volume in vivo. Guan and colleagues employed a straightforward high-temperature pyrolysis method to prepare nanoscale microporous carbon materials (CNPs) from nanoscale covalent organic frameworks (COFs) precursors. Research indicated that the PCE of CNPs in the NIR-II region reaches 50.6% [64]. In in vitro experiments, under 1064 nm laser irradiation and at a CNP concentration of up to 200 mg/mL, the viability of human breast cancer cells and human acute T-lymphoblastic leukemia cells decreased to 17.2 ± 1.9% and 39.2 ± 3.7%, respectively, thereby demonstrating their tumor ablation capability.

In terms of biological applications for the in vivo reduction in tumor volume, Zhao et al. developed water-dispersible nanoparticles incorporating two nanographene-porphyrin hybrids (NGP-1-NPs and NGP-2-NPs). These nanoparticles exhibit strong absorption in the NIR windows, achieving high photothermal conversion efficiencies of 69% [65]. Moreover, treatment with NGP-2-NPs under laser irradiation resulted in the complete elimination of tumors without recurrence over the following 14 days, thereby demonstrating the promising photothermal therapeutic efficacy of NGP-2-NPs.

**Figure 7:**
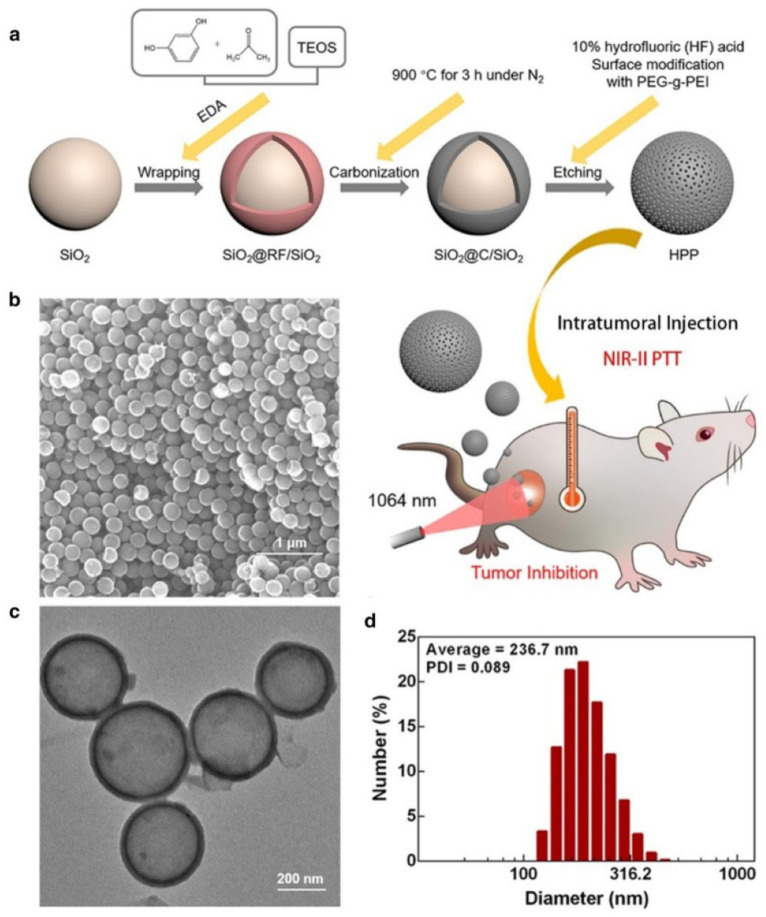
Synthesis and characterization of HPP. (**a**) Schematic illustration of the preparation processes of HPP and its application for cancer photothermal therapy. (**b**) SEM image of HPP; (**c**) TEM image of HPP. (**d**) Hydrodynamic diameter distribution of HPP. Reprinted with permission from ref. [66].

Additionally, the lower the radiation exposure required for PTT in the NIR-II region, the greater the biological safety of the treatment. Xu et al. synthesized a hollow carbon nanosphere modified with polyethylene glycol-graft-polyethylenimine (HPP) that serves as an NIR-II-responsive PTA (Figure 7a) [66]. The average diameter size of HPP is 236.7 nm with a large internal cavity (Figure 7b–d). Due to its uniform morphology, stable structure, and distinctive absorption in the NIR-II region, a remarkable heat conversion efficiency of 45.1% was achieved under 1064 nm laser irradiation. HPP demonstrated the capability to generate sufficient heat at a safe power density of 0.6 W/cm^2^ with a low concentration of 10 µg/mL, indicating that this material is an ideal candidate for safe and effective PTT.

#### 2.1.4. Quantum Dots

Quantum dots (QDs), defined as zero-dimensional nanostructures exhibiting three-dimensional quantum confinement, have emerged as pivotal materials in biomedical applications [67]. Among these, graphene quantum dots (GQDs) are particularly notable due to their exceptional biological properties, including high water solubility, stability, and biocompatibility [68,69,70]. A significant advancement was reported by Liu et al., who synthesized 9T-GQDs via a one-step solvothermal method utilizing phenol precursors and hydrogen peroxide under a 9T external magnetic field [71]. These GQDs demonstrated remarkable tumor suppression capabilities when subjected to 1064 nm laser irradiation (1.0 W/cm^2^), effectively killing tumor cells in vitro and inhibiting tumor growth in vivo. Their efficacy arises from two synergistic mechanisms: the enhanced permeability and retention (EPR) effect [72], which facilitates nanoparticle accumulation in tumor tissues, and nanoparticle-induced endothelial leakiness (NanoEL) [73,74,75,76,77], which increases vascular permeability at tumor sites. These properties position 9T-GQDs as promising agents for PTT in the near-infrared II (NIR-II) window.

**Figure 8:**
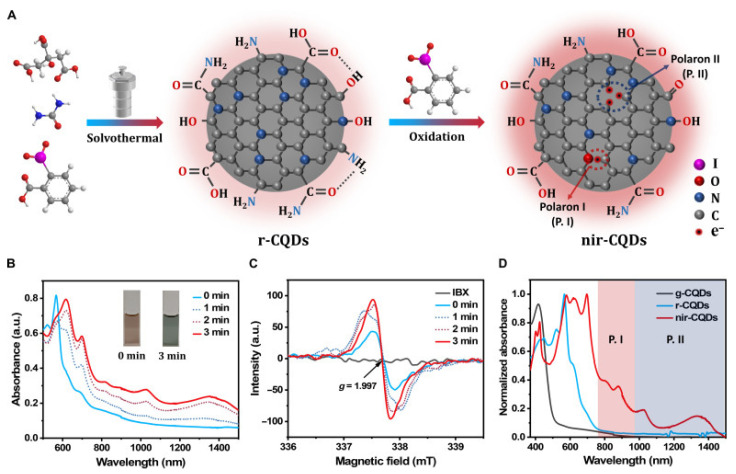
Schematic of the synthesis of r-CQDs and nir-CQDs. (**A**) Schematic of the synthesis of r-CQDs and nir-CQDs. In situ (**B**) absorbance and (**C**) ESR spectra of r-CQDs during treatment with IBX in DMSO; inset: photographs of r-CQD DMSO solution without and with IBX treatment for 5 min. (**D**) Normalized absorbance spectra of g-CQDs, r-CQDs, and nir-CQDs in DMSO at 25 μg mL^−1^. Reprinted with permission from ref. [83].

Concurrently, carbon quantum dots (CQDs) have attracted attention as biocompatible organic semiconductors with tunable bandgaps and versatile photophysical properties, including photoluminescence and photothermal conversion [78,79,80,81,82]. Despite advancements, achieving precise control over optical bandgaps in the NIR-II region remains a significant challenge. In response, Zhang et al. developed nonmetallic CQDs with tailored NIR-II absorption bands through a polaron engineering strategy [83]. The CQDs achieved a PCE of 40% under 1064 nm irradiation, enabling highly effective PTT in the NIR-II window (Figure 8). Additionally, Ren et al. developed a theranostic nanoplatform, Cet-CDs-SNO (CCS), to facilitate multimodal imaging-guided synergistic therapy for colon cancer [84]. The CCS exhibited excellent PCE (31.8%), photostability, and biocompatibility in the context of colon cancer treatment. In addition, in vitro and in vivo experiments have demonstrated that CCS exhibits exceptional tumor-targeting capabilities, attributed to the assembly of cetuximab.

### 2.2. Organic Agents

Organic photothermal agents (OPTAs) offer superior biocompatibility compared to inorganic photothermal agents (IPTAs), as they avoid the toxic effects associated with non-releasing inorganic ions. This makes OPTAs particularly advantageous for addressing long-term biosafety concerns. In the following, we will introduce polymer-based photothermal agents (PPTAs) and small-molecule photothermal agents (MPTAs) [24,85].

#### 2.2.1. Semiconductor Polymers

Semiconducting polymers have emerged as crucial materials in PTT due to their unique structural and performance advantages [86]. These macromolecules exhibit tunable optical, electrical, and thermal properties through π-electron delocalization systems (based on mechanisms such as electron hopping and tunneling effects). The alternating arrangement of electron-donating (donor) and electron-accepting (acceptor) units in their molecular structure endows them with exceptional light absorption capabilities in the NIR window [87,88]. Compared to inorganic nanomaterials, semiconducting polymers not only demonstrate superior PCE but also exhibit excellent photostability and biocompatibility [89]. Cao et al. reported a thieno-isoindigo derivative-based donor–acceptor (D-A) polymer (PBTPBF-BT) with a very low HOMO–LUMO gap of 1.24 eV, exhibiting a maximum absorption peak at approximately 1107 nm [90]. NP_PBTPBF-BT_ showed high mass extinction coefficients (83.9 mL/cm·mg), superior PCE (66.4%), and excellent photostability under irradiation with a 1064 nm laser, resulting in high efficiency in ablating cancer cells. Wei et al. used an emulsion method to synthesize a new type of diketopyrrole polymer (DPP-IID-FA), which exhibited strong light absorption capabilities and excellent photothermal properties in the NIR-II regions [91]. The PCE in the NIR-II light region reached 49.5%. After five cycles of heating and cooling, the DPP-IID-FA maintained its temperature-increasing ability, demonstrating that the as-prepared nanoparticles possess excellent photostability.

In addition to their photothermal properties, these materials also exhibit selective tumor ablation capabilities. A notable example is the organic photothermal nanoagent SPN_I–II_, as reported by Jiang et al., which primarily consists of the semiconducting copolymer poly [(diketopyrrolopyrrole-cyclopentadithiophene)-ran-(diketopyrrolopyrrole-thiadiazoquinoline)] (PDCDT) [92]. SPN_I–II_ nanoparticles demonstrated dual absorption peaks in the NIR-I and NIR-II windows, achieving a PCE of 43.4% under 1064 nm laser irradiation. Under NIR-II light, SPN_I–II_-mediated PTT facilitated precise tumor ablation while preserving normal tissues. Collectively, these studies systematically illustrate the multifaceted advantages of semiconducting polymers in precision oncology.

#### 2.2.2. Organic Molecules

Organic molecule PTAs remain challenging due to their synthesis difficulty and photothermal stability (Table 2). Indocyanine green (ICG) is a widely utilized organic molecule PTA, notable for its significant absorption peak in the NIR region [93]. Importantly, ICG is the only near-infrared absorbing dye currently approved by the U.S. Food and Drug Administration (FDA) [94]. However, the poor thermal stability of ICG significantly limits its application in PTT [95]. Therefore, researchers put their effort into developing ICG derivatives with good thermal stability and a high PTCT in the NIR-II window. Zhao et al. combined indole salts with polymethyl through the Knoevenagel reaction and synthesized six polymethylcyanines with larger conjugated structures (IC-790, IC-830, IC-1030, IC-1060, IC-1080, and IC-1224) [96]. Among these derivatives, IC-1224 demonstrates a PCE of 83.2% in the NIR-II window, with an absorption wavelength exceeding 1200 nm, showcasing excellent properties as a PTA.

Polypyrrole (PPy) nanomaterials have garnered significant attention due to their high conductivity, excellent stability, good biocompatibility, and ease of synthesis. These materials possess considerable potential as photothermal coatings for various PTT, offering a promising approach to enhance nanoscale PPy-functionalized coatings in medical systems [97]. The electronic structure and associated optical properties of PPy can be finely tuned through a controlled doping process [98,99,100]. As the doping level increases, polariton bands and dual polariton bands emerge within the band gap of PPy, leading to strong light absorption in the NIR-II region [99,101]. These characteristics render PPy highly promising for applications in the biomedical field. Wang et al. developed ultrathin PPy nanosheets using a space-confined synthesis method with layered FeOCl as the removable template [98]. The formation of bipolarons in highly doped PPy nanosheets results in unique broadband absorption, with an extinction coefficient reaching up to 27.8 L/g·cm at 1064 nm, making these nanosheets suitable as efficient photothermal agents in the NIR-II window. Under 1064 nm laser irradiation, the PCE of ultrathin PPy nanosheets can reach 64.6%, surpassing previously reported PTAs active in the NIR-II window. Both in vitro and in vivo studies demonstrate that these ultrathin PPy nanosheets exhibit good biocompatibility and notable tumor ablation ability in the NIR-II window. This research highlights the potential of ultrathin two-dimensional polymers with unique optical properties for biomedical applications.

Conjugated small molecules, characterized by their electron-delocalized structures and light-harvesting capabilities, have recently demonstrated significant advantages in light absorption and amplification. Li et al. engineered D-π-A-π-D conjugated small molecules (IR-TT, IR-TS, and IR-SS) using a Se-tailoring strategy to red-shift the light-harvesting peak from the NIR-I to the NIR-II window [102]. The IR-SS molecule has an absorption peak at 1060 nm, and its nanoparticle shows a high photothermal energy conversion efficiency of 77%. Notably, almost 95% of human lung cancer cells were killed at the IR-SS NPs concentration of 50 µg/mL upon 1064 nm laser irradiation (1 W/cm^2^) for 5 min.

**Table 1:** Summary of the properties and applications of representative NIR-II PTT materials discussed in this review (Ex, excitation wavelength (nm); PCE, photothermal conversion efficiency (%); ΔT, temperature increase (°C); NA, not applicable).

Type	Nanoparticles /Nano-Conjugates	Ex	PCE	Application	Ref.
Metal nanomaterials	Au-on-AuNR hybrid structures: structure 2a–2d and nanocorals 3b–3d	1060	26.1, 26.7, 25.6, 26.6, 56.9, 67.2 and 59.8	/	[28]
	Au@Cu2−xS core@shell NCs	1064	43.25	HeLa cells	[29]
	GNR@SiO_2_@MnO_2_	1064	27.47	U87MG cells, U87MG-tumor-bearing mice	[30]
	AuHNRs	1064	33	SCC-7 cells, SCC-7 tumor-bearing nude mice	[36]
	M-AuHNRs	1064	34	HeLa, murine breast cancer 4T1, HepG-2, and COS-7 cells, HepG-2 tumor-bearing nude mice	[37]
Metal sulfide/oxide nanomaterials	Ni_9_S_8_	400–1100	46 (1064)	HeLa cells, HeLa tumor-bearing mice	[41]
	Cu_2_MnS_2_	800–1300	49.38 (1064)	MCF-7 and HeLa cells, S180 tumors-bearing BALB/c nude mice	[42]
	CuS@PDA/Pd	1064	50.6	MCF-7, 4T1, MDA-MB-231, HepG2, and B16F10 cells, 4T1 tumor-bearing mice	[43]
	CuCo_2_S_4_-Pt-PEG	1064	78.46	4T1 cells, 4T1 tumor-bearing mice	[46]
	RCuS@tMCP	1064	69.6	RAW264.7 and 4T1 cells, 4T1 tumor-bearing mice	[48]
	BCS NPs	1064	29.8	4T1 cells, 4T1 tumor-bearing mice	[50]
	AuDAg_2_S	1064	67.1	HUVEC, hepatic and CT26 colon tumor cells, CT26 tumor-bearing nude mice	[51]
	AT-CuS NCs	1064	94.3	U87 cells	[52]
	HMNC	1064	36.3	HeLa cells, HeLa tumor-bearing mice	[57]
	AuNCs@SiO_2_	1064	82.2	4T1 and A549 cells, 4T1 tumor-bearing BALB/c nude mice	[58]
	H-SiOx	1064	48.6	4T1 cells, 4T1 tumor-bearing mice	[59]
Carbon-based nanomaterials	CNPs	1064	50.6	MCF-7 cells and Jurkat cells, MCF-7 tumor-bearing mice	[64]
	Water-dispersible nanoparticles containing two nanographene-porphyrin hybrids (NGP-1-NPs and NGP-2-NPs)	808, 1064	60, 69	4T1 and MCF-7 cells, 4T1 tumor-bearing mice	[65]
	HPP	1064	45.1	4T1 and MCF-7 cells, 4T1 tumor-bearing mice	[66]
Quantum dots	9T-GQDs	1064	33.45	4T1, HeLa and NCI–H196 cells, 4T1 tumor-bearing mice	[71]
	nir-CQD	1064	40	4T1 cells, 4T1 tumor-bearing mice	[83]
	Cet-CDs-SNO	1064	31.8	HCT-116 cells, HCT-116 xenograft tumor-bearing nude mice	[84]
Semiconductor polymers	NP_PBTPBF-BT_	1064	66.4	MDA-MB-231 cells, MDA-MB-231 tumor-bearing mice	[90]
	DPP-IID-FA	1064	49.5	HeLa cells, tumor xenografts in nude mice	[91]
	SPN_I–II_	808, 1064	44.9, 43.4	4T1 cells, 4T1 xenograft tumor-bearing nude mice	[92]
Small organic molecules	IC-790, IC-830, IC-1030, IC-1060, IC-1080 and IC-1224	1064	83.2 (IC-1224)	4T1 cells, 4T1 tumor-bearing mice	[96]
	Ultrathin PPy nanosheets	1064	64.6	MDA-MB-231 cells, MDA-MB-231 xenograft-bearing mice	[98]
	IR-TT, IR-TS, and IR-SS	1064	61, 73, and 77	A549 and 4T1 cells, 4T1-tumor-bearing mice	[102]

**Table 2:** Molecular characteristics of organic molecules.

Molecular Name	Chemical Structural Formula	Constitutional Formula
ICG	C_43_H_47_N_2_NaO_6_S_2_	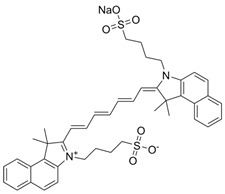
Polypyrrole	C_4_H_5_N	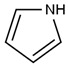
Conjugated small molecules (IR-SS)	C_74_H_100_N_2_S_2_Se_2_	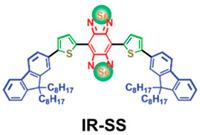

## 3. PTT-Based Synergy Therapy

### 3.1. Combination of PTT and PDT

PDT is a treatment strategy that relies on the interaction between light, molecular oxygen (O_2_), and a photosensitizer (PS). Upon irradiation with NIR light, the PS absorbs photons and transitions to an electronically excited singlet state. This singlet state can undergo intersystem crossing to form a long-lived triplet state, which may release energy through fluorescence, heat, or other photophysical processes. More importantly, the excited triplet state facilitates the production of reactive oxygen species (ROS) through two distinct mechanisms. These ROS play a crucial role in selectively destroying tumor cells [103,104,105]. However, hypoxic conditions in deep-seated tumors, often located far from blood vessels, significantly limit the efficacy of PDT.

To address the challenges posed by hypoxia, PTT serves as a complementary approach [106]. By generating localized heat, PTT can increase blood flow within tumor tissues, thereby improving oxygen delivery and alleviating hypoxia to enhance PDT efficacy. Furthermore, the oxygen-independent heating effect of PTT can directly eliminate hypoxic tumor cells that are resistant to PDT. To integrate PDT and PTT into a single treatment platform, various nanomaterials with NIR-induced photothermal conversion properties have been developed as carriers for photosensitizers. These nanocarriers—such as graphene oxide [107], gold nanostructures [108,109], CuS nanomaterials [110], and polydopamine [111], enable synergistic therapeutic effects by combining the benefits of PDT and PTT.

Among emerging advances, high-efficiency NIR-II type-I PDT/PTT systems have been developed to address the limitations of hypoxic tumor environments. For instance, Wen et al. synthesized and characterized three donor-acceptor semiconducting polymers based on chalcogen elements: PTS, PTSe, and PTTe. These polymers demonstrated strong NIR-II absorption properties, with PTTe NPs exhibiting superior PCE and enhanced ROS generation (specifically superoxide anion radicals, O_2_^−^) under 1064 nm laser irradiation. This resulted in exceptional therapeutic performance in both in vitro and in vivo settings. Similarly, Bian et al. developed a series of NIR-II dyes (BHs) by incorporating a rigid xanthonium moiety into the conjugation core of cyanine dyes [112]. Among these, BH 1024 exhibited the best singlet oxygen generation capability and moderate photothermal heating under 1064 nm irradiation. In cellular experiments, BH 1024 nanoparticles induced 92.8% cell death through the combined effects of ROS production and localized hyperthermia. In contrast, standalone PTT and PDT achieved only 68.2% and 23.1% cell death, respectively, highlighting the superior efficacy of the combined therapeutic approach.

In another study, Tian et al. synthesized carbon nitride nanoparticles (CN-NPs) through the copolymerization of perylene-3,4,9,10-tetracarboxylic dianhydride (PTCDA) with melem at elevated temperatures [113]. These CN-NPs demonstrated dual capabilities: converting photons into heat (~47.6 °C) and generating ROS under single 1064 nm laser irradiation (1 W/cm^2^). The synergistic effects of PTT and PDT were evidenced by high cell death rates observed in vitro. Furthermore, CN-NPs effectively treated large solid tumors with no recurrence observed within 14 days post-treatment. Additionally, Gao et al. developed oxygen-deficient black tin oxide nanoparticles (SnO_2−x_@SiO_2_-HA) as multifunctional therapeutic agents [114]. These nanoparticles exhibited both photothermal and ROS-generating properties under 1064 nm laser irradiation, effectively inhibiting the growth of mouse breast cancer cells. The strong affinity between hyaluronic acid (HA) and the CD44 protein facilitated the selective uptake of SnO_2−x_@SiO_2_-HA nanoparticles by CD44-overexpressing tumor cells, enabling precise targeting and treatment.

These advancements highlight the potential of combining PDT and PTT in a single nanoplatform, leveraging NIR-II-responsive materials to overcome the limitations of traditional therapies. The development of multifunctional nanomaterials paves the way for innovative and effective cancer treatments, particularly in hypoxic and deep-seated tumor environments.

### 3.2. Combined PTT and Immunotherapy

Cancer immunotherapy leverages the body’s natural defense mechanisms to identify, attack, and eliminate cancer cells. Currently, innovative approaches such as immune checkpoint blockade (ICB) strategies [115,116,117,118], chimeric antigen receptor T-cell (CAR-T) therapies [119,120,121,122], and cancer vaccines [123] have made significant progress. However, these methods face challenges, including low immune response rates and limited anti-tumor efficacy [124,125,126]. To address these limitations, combining PTT with immunotherapy has shown great promise in enhancing tumor treatment [127,128]. In 2001, Morita et al. observed that preoperative hyperthermia increased lymphocyte infiltration around cancer lesions [129]. This finding paved the way for the realization that hyperthermia-induced tumor ablation could release tumor-associated antigens (TAAs), which are recognized by dendritic cells (DCs) and presented to T-cell receptors, especially when paired with immunoadjuvants [130,131,132]. Additionally, blocking immune checkpoints can further enhance T-cell-mediated immune responses [133,134,135,136]. Thus, hyperthermia-ablated tumors can serve as an in situ autologous cancer vaccine when combined with immunotherapy, offering immense potential to eradicate both primary and metastatic tumors [137,138,139,140]. Fan et al. introduced a novel approach to convert immunogenically dying tumor cells into a versatile cancer vaccine platform [141]. This whole-tumor cell vaccine effectively promoted antigen-presenting cell (APC) activation and antigen presentation, eliciting strong anti-tumor immune responses in murine models of melanoma and colon carcinoma. When combined with ICBs, this approach demonstrated remarkable therapeutic potential, achieving complete tumor regression and long-term protection against recurrence in approximately 78% of tumor-bearing animals.

Hyperthermia ablation of tumors can result in cancer cell death in an immunogenic manner [141,142]. For instance, Xiong et al. developed biomimetic nanoplatforms (bmNPs) by camouflaging PTAs with T-cell membranes [143]. Under NIR-II laser irradiation, these platforms mediated a photothermal effect that directly ablated tumors and released damage-associated molecular patterns (DAMPs), thereby inducing immunogenic cell death (ICD). Similarly, Huang et al. designed defect-rich MoSe_2_-DPEG nanomaterials that efficiently performed NIR-II PTT and enhanced oxidative stress by depleting intracellular glutathione (GSH) [144]. This strategy induced ICD, improved responses to checkpoint blockade immunotherapy (CBI), and stimulated CD8^+^ T-cell-mediated systemic antitumor immunity, effectively suppressing tumor growth and metastasis. Chen et al. introduced a pH-sensitive NIR-II photothermal liposome nanocomplex (LNCDS series) with photocontrolled release capabilities [145]. Upon exposure to a 1064 nm laser, the PTT effect ruptured the liposomes, releasing cytotoxic enzymes (DNase I) and immunostimulants (SIS3). DNase I induced cancer cell death and triggered immune cell pyroptosis, while SIS3 further activated natural killer (NK) cells and CD8^+^ cytotoxic T lymphocytes (CTLs). This combination therapy inhibited both primary and distant tumor growth and reduced the progression of lung metastasis in a 4T1 breast cancer mouse model. Moreover, PTT can enhance tumor sensitivity to immunotherapy by reversing the immunosuppressive tumor microenvironment (ITME) [146,147]. Zhang et al. utilized natural melanin nanoparticles derived from cuttlefish ink to coat macroporous mesoporous SiO_2_ and loaded azobisisobutylimidazoline hydrochloride (AIPH@MS) [148]. Under 1064 nm laser irradiation, the heat generated induced direct tumor cell death, releasing AIPH and generating oxygen-independent alkyl free radicals for additional tumor cell damage. This approach reprogrammed tumor-associated macrophages (TAMs) from an M2 to an M1 phenotype. When combined with anti-PD-1 therapy, this strategy awakened suppressed immune responses, effectively suppressing both primary and metastatic tumors [145].

Ma et al. engineered photothermal converters by self-assembling AuNPs on fluid liposomes [149]. Under near-infrared II (NIR-II) laser irradiation, these converters induced tumor cells to release DAMPs such as ATP, calreticulin (CRT), and the high-mobility group box 1 protein (HMGB1). These molecules acted as immunostimulatory signals, promoting phagocytosis by DCs and activating T cells to trigger potent anti-tumor immune responses. In vivo, NIR-II PTT effectively induced ICD in deep tumor tissues, activating both innate and adaptive immune responses. When combined with α-PD-1 therapy, this approach significantly enhanced long-term tumor control and inhibited distant metastases.

In conclusion, the integration of PTT with immunotherapy not only improves the efficacy of immune checkpoint inhibitors but also stimulates systemic anti-tumor immunity. These synergistic strategies possess substantial potential for enhancing cancer treatment outcomes.

### 3.3. Combined PTT and Chemotherapy

Chemotherapy, a classical clinical approach for treating both primary and metastatic tumors, primarily utilizes cytotoxic agents such as doxorubicin, paclitaxel, and cisplatin to induce apoptosis or necrosis in tumor cells [150,151]. However, its efficacy is limited by two critical challenges: first, the rapid systemic clearance and nonspecific distribution of these drugs result in systemic toxicity and inadequate local therapeutic concentrations at tumor sites; second, prolonged treatment can lead tumor cells to develop multidrug resistance (MDR) mechanisms, which significantly contribute to chemotherapy failure in clinical practice [152]. To overcome these limitations, researchers have proposed a synergistic strategy that combines PTT with chemotherapy. Nanomaterial-based drug carriers enable precise tumor accumulation through active targeting (via surface-conjugated ligands) or passive targeting (via the EPR effect) [153]. Localized hyperthermia induced by NIR light irradiation dilates tumor vasculature and enhances vascular permeability, thereby significantly improving the delivery efficiency of chemotherapeutic drugs to deep tumor tissues [154]. For example, Yu et al. developed pH-sensitive doxorubicin (DOX)-conjugated block copolymer nanoparticles (PADD@SPs), where the photothermal effect promotes drug penetration into tumor tissue [155]. The micellar core stabilizes drug loading under physiological conditions while facilitating on-demand drug release in the acidic lysosomal microenvironment of tumor cells.

The stimuli-responsive properties of nanocarriers enable spatiotemporally controlled drug release. Sun et al. synthesized a porous bimetallic Au@Pt core-shell nanostructure using the polymer micelle template method, subsequently incorporating DOX into the mesopores of the Au@Pt nanostructure with the aid of phase change materials (PCM). This process ultimately led to the formation of an Au@Pt-DOX-PCM-PEG nanotherapeutic drug designed for NIR-II activated chemotherapy [156]. The nanocomposite exhibited significant synergistic effects when combined with NIR-II PTT and chemotherapy. Specifically, the material generates oxygen by catalyzing endogenous hydrogen peroxide (H_2_O_2_) present in the tumor, which not only alleviates tumor hypoxia by directly supplying oxygen but also promotes the release of the chemotherapy drug DOX through the photothermal effect, thereby enhancing the efficacy of chemotherapy. Furthermore, NIR-II PTT contributes to the elimination of cancer cells. This combined treatment strategy effectively addresses the limitations of single treatment modalities, significantly improving therapeutic outcomes while reducing the side effects associated with chemotherapy drugs. Zhang et al. developed AuHNR@MnO_2_@CS (AuMC), which is responsive to the NIR-II window [157]. This material was constructed through a one-step sequential coating of MnO_2_ and chitosan (CS) layers onto AuHNRs, exhibiting strong LSPR. In the tumor microenvironment (TME), the overexpression of GSH specifically triggers the degradation of the MnO_2_ layer, generating three synergistic effects: (1) the PCE at 1064 nm significantly increases from 25.4% to 33.2%, representing a 30% enhancement; (2) released Mn^2+^ ions amplify chemodynamic therapy (CDT) through Fenton-like reactions; and (3) the CS outer layer regulates the sustained release kinetics of Mn^2+^ ions. This dual-responsive mechanism synergizes PTT with CDT, achieving TME-activated therapeutic amplification (24.6% PCE improvement) while substantially enhancing both treatment precision and antitumor efficacy.

The synergistic strategy of combining chemotherapy with PTT not only enhances local therapeutic efficacy but also reduces systemic toxicity through microenvironment-responsive regulation. This approach marks a significant advancement in the field of precision oncology.

## 4. Clinical Progress of PTT

**Figure 9:**
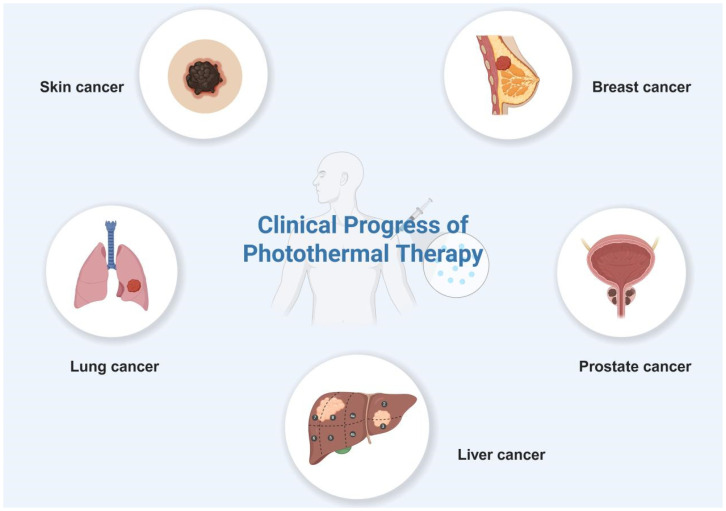
Clinical progress of photothermal therapy in tumors.

For decades, the development of effective cancer therapeutics has been a major focus of biomedical research. Conventional approaches, such as surgery, chemotherapy, and radiotherapy, remain foundational treatments; however, they are frequently associated with significant side effects and suboptimal therapeutic outcomes, particularly in advanced malignancies [158,159]. These limitations have driven the exploration of alternative strategies, among which PTT has emerged as a promising modality, supported by growing preclinical and clinical validation [24,160,161]. The advancement of nanomedicine has further accelerated PTT development, with numerous nanotherapeutic agents either approved for clinical use or currently undergoing trials. While nanomaterial-mediated PTT demonstrates unique advantages in tumor targeting and localized energy conversion, its clinical translation faces substantial challenges. Most PTT systems rely on laser devices that utilize endogenous tissue chromophores for thermal ablation, a mechanism that limits treatment depth and precision [162]. Furthermore, despite extensive preclinical studies demonstrating tumor suppression in animal models [163,164,165,166], only a limited number of PTT protocols have progressed to human trials. To date, clinical applications of PTT have primarily been explored in specific tumor types. Here, we introduce several tumors for which clinical trials of PTT have been conducted (Table 3 and Figure 9).

### 4.1. Skin Cancer

Skin cancers can be classified into two main types based on their cell of origin: melanoma skin cancer, which arises from melanocytes, and non-melanoma skin cancer, which originates from keratinocytes. Non-melanoma skin cancers are further categorized into basal cell carcinoma (BCC) and squamous cell carcinoma (SCC) according to their severity [167]. Although non-melanoma skin cancers account for 95% of all reported skin cancer cases (75% BCC and 20% SCC), the majority of skin cancer-related deaths are attributed to melanoma, with mortality rates reaching as high as 80% [168]. Common treatments for early-stage skin cancer include excisional surgery, Mohs surgery, radiation therapy, curettage and electrodesiccation, cryotherapy, and PDT [169,170,171,172,173]. However, these conventional treatments often demonstrate limited efficacy and significant side effects in patients with locally advanced disease. In 1997, Yanovsky et al. introduced in situ photoimmunotherapy (ISPI), a method that combines targeted PTT with immunotherapy [173]. Li et al. conducted a preliminary clinical study involving 11 patients with metastatic melanoma to evaluate the safety and efficacy of ISPI, utilizing imiquimod as an immunomodulator in patients with advanced melanoma [174]. The study results indicated that lesions responded to ISPI in all treated areas, with eight lesions achieving a complete local response (CLR). CLR was also noted in lesions in non-treated areas in four patients. Among the patients studied, the probability of overall survival at 12 months was 70%. These findings suggest that the ISPI approach with imiquimod is both safe and well-tolerated in patients with metastatic melanoma.

### 4.2. Prostate Cancer

Prostate cancer is the second most diagnosed malignancy in men and the fifth leading cause of death worldwide [168]. The mortality rate from prostate cancer increases with age, being particularly prevalent in older men with an average age of 66. While prostate cancer is typically asymptomatic in its early stages, symptoms such as frequent urination, urgency, and dysuria may manifest in advanced stages. Following metastasis, patients may experience urinary retention and bone pain. The most commonly employed treatments for prostate cancer include surgery, chemotherapy, and radiotherapy, which demonstrate good efficacy in the initial stages of the disease [175]. However, as the disease progresses, these treatments may become less effective and can lead to various side effects. Post-prostatectomy, patients may encounter complications such as urinary retention, urinary incontinence, and erectile dysfunction. Additionally, chemotherapy can result in adverse effects such as hair loss, weakness, pain, and difficulty breathing, whereas radiation therapy may damage healthy tissue due to targeting challenges [176,177,178]. Consequently, researchers are actively exploring new treatment options. Rastinehad et al. reported on 16 patients diagnosed with low- or intermediate-risk localized prostate cancer (stage T2a or less) who underwent PTT [179]. In this study, laser-driven gold silica nanoshells (GSNs) were utilized in conjunction with magnetic resonance-ultrasound fusion imaging technology for the local ablation of low- and intermediate-grade tumors within the prostate. The results indicated that 94% (15/16) of the patients successfully achieved GSN-mediated focal laser ablation, and 87.5% (14/16) of the lesions in the ablation area were tumor-negative after 12 months. Furthermore, no significant differences were observed in the International Prostate Symptom Score or the Male Sexual Health Scale post-treatment.

### 4.3. Breast Cancer

Although advances in early diagnosis and treatment have significantly reduced breast cancer mortality over the past few decades, it remains one of the leading causes of cancer-related deaths among women worldwide. According to the latest data from the American Cancer Society, breast cancer constitutes 31% of all new cancer diagnoses in women, while lung cancer accounts for only 13% [180]. Surgery is the primary method of breast cancer treatment, involving the complete removal of the primary tumor and assessment of lymph node involvement [181]. In addition to surgery, breast cancer can also be managed with chemotherapy [182], radiotherapy [183], endocrine therapy [184], targeted therapy [185], immunotherapy [186], and newly developed neoadjuvant therapy [187]. The choice of treatment primarily depends on the patient’s clinical stage and tumor type. Existing treatments exhibit certain limitations and side effects, including suboptimal efficacy, lack of specificity, and potential damage to normal organ function. Therefore, individualized treatment plans must be developed for patients with different breast cancer subtypes. PTT presents a promising alternative or adjunctive treatment option for breast cancer. Li et al. recruited 10 patients with advanced (stage III or IV) breast cancer and employed 805 nm laser irradiation to enhance the thermal effect through local injection of the light-absorbing agent ICG, in conjunction with the immune enhancer glycated chitosan (GC) to stimulate the immune response [188]. In this study, the objective effective rate reached 62.5% (including one complete remission and four partial remissions), and the clinical benefit response rate was 75%. Schwartzberg et al. recruited 61 patients with invasive breast cancer to undergo laser treatment prior to tumor resection. Pathological analysis confirmed complete tumor ablation in 51 cases (84%) [189]. The results of this research indicate that PTT holds significant potential for clinical application in breast cancer treatment.

### 4.4. Liver Cancer

Liver cancer is a prevalent tumor of the digestive system and ranks as the sixth most common cancer worldwide [190]. Treatment options encompass radical surgery [191], molecular-targeted therapy [192], and neoadjuvant therapy [193]. However, patients with advanced liver cancer face limited treatment alternatives and a dismal prognosis, with a 5-year survival rate of merely 18% [194]. The toxicity and adverse reactions associated with anti-tumor drugs have prompted researchers to continually explore novel treatment strategies. Vogl et al. conducted magnetic resonance imaging-guided laser interstitial thermal therapy (LITT) on 603 patients (mean age 61.2 years) with liver metastases originating from colorectal cancer [195]. Within a 6-month follow-up period, the local recurrence rate of metastatic tumors in treated patients was recorded at 1.2% to 4.4%, with an average survival duration of 4.4 years. Pacella et al. reported that among 148 patients (144 with biopsy-confirmed hepatocellular carcinoma) treated with 1064 nm laser ablation, the long-term survival rates at 1, 3, and 5 years were 89%, 52%, and 27%, respectively, achieving an overall complete lesion ablation rate of 82% [196]. In another cohort of 74 patients with biopsy-confirmed small cell liver cancer (tumor diameter range: 0.8–4.0 cm) who underwent percutaneous laser thermal ablation, the overall survival rates at 1, 3, and 5 years were 99%, 68%, and 15%, respectively [197]. These findings underscore the significant role of PTT in the management of liver cancer, potentially offering patients improved prognoses and survival opportunities.

### 4.5. Lung Cancer

Lung cancer has emerged as the malignant tumor with the highest morbidity and mortality worldwide. It is the leading cause of cancer-related morbidity and mortality in men and the second leading cause in women [198]. Lung cancer is primarily classified into two types: small cell lung cancer (SCLC) and non-small cell lung cancer (NSCLC), with NSCLC being the most prevalent subtype, accounting for 85–90% of all lung cancer cases. NSCLC comprises several histological subtypes, including lung adenocarcinoma (LUAD), lung squamous carcinoma, and large cell lung cancer [199]. For stages I and II of NSCLC, treatment primarily involves surgical resection of the tumor, supplemented by adjuvant therapy [200,201]. When the disease progresses to stages III or IV, treatment strategies typically transition to chemotherapy or radiotherapy. In cases where lung cancer invades the bronchus and leads to obstructive endobronchial cancer, existing treatments often fail to alleviate acute symptoms promptly. LITT is a treatment that typically involves the placement of laser fibers within tumors and has been investigated for various cancer indications. Li et al. introduced a method of central airway thermal ablation, aimed primarily at palliatively relieving airway obstruction and achieving curative effects in certain cases [162]. Preoperative evaluation utilized CT scans and bronchoscopy to confirm the extent of the disease, while laser treatment could rapidly eliminate airway tumors and significantly enhance lung function. This innovative treatment modality has provided new therapeutic options for lung cancer patients.

**Table 3:** Examples of tumors treated clinically with photothermal therapy.

Type	Treatment	Nanoparticles/Nano-Conjugates	Number of Patient Enrolled	Ref.
Skin cancer	Combined photothermal therapy and immunotherapy	Topical imiquimod and indocyanine green	11	[174]
Prostate cancer	Photothermal therapy	Laser-driven gold silica nanoshells	16	[179]
Breast cancer	Combined photothermal therapy and immunotherapy	Indocyanine green combined with glycated chitosan	10	[188]
	Laser treatment	-	61	[189]
Liver cancer	Laser interstitial thermal therapy (LITT)	-	603	[195]
	Laser interstitial thermal therapy (LITT)	-	148	[196]
	Laser interstitial thermal therapy (LITT)	-	74	[197]
Lung cancer	Laser interstitial thermal therapy (LITT)	-	-	[162]

## 5. Conclusions

With the in-depth research into precision medicine, NIR-II-PTT has shown promising application prospects across various scenarios. The combination of NIR-II-PTT with PDT, chemotherapy, and immunotherapy has significantly expanded the potential applications of PTAs, particularly those operating within the NIR-II window, for tumor treatment. As research progresses on near-infrared second-region photothermal agents, clinical researchers will have access to an increasing number of viable and reliable photothermal agents, enabling them to investigate their applicable scenarios and therapeutic effects in clinical settings. During this process, clinical researchers will also articulate more detailed requirements for near-infrared two-region photothermal agents, thereby promoting their development. In summary, photothermal agents in the near-infrared second region exhibit very promising prospects in photothermal therapy and in the synergistic treatment of photothermal therapy with other modalities. Future interdisciplinary research involving nanomaterials and clinical medicine holds significant potential.

## Abbreviations

WHO	World Health Organization
PTT	Photothermal therapy
PTAs	Photothermal agents
NIR	Near-infrared
MPE	Maximum permissible exposure
ANSI	American National Standards Institute
NIR-II	Second near-infrared
PCE	Photothermal conversion efficiency
AuNPs	Gold nanoparticles
SPR	Surface plasmon resonance
LSPR	localized surface plasmon resonance
GSM	GNR@SiO_2_@MnO_2_
AuHNRs	Hollow gold nanorods
M-AuHNRs	Microscale hollow gold nanorods
CuS	Copper sulfide
Ag_2_S	Silver sulfide
PbS	Lead sulfide
FeS	Iron sulfide
5FU	5-fluorouracil
CuAAC	Copper-catalyzed azide-alkyne cycloaddition
CuS NPs	Copper sulfide nanoparticles
RBCm	Red blood cell membrane
tMCP	CpG/protamine
NCS	Non-stoichiometric copper sulfide
BCS NPs	Biomineralized copper sulfide nanoparticles
SERS	Surface-enhanced Raman scattering
2D	two-dimensional
AT-CuS NCs	Atomic-thin 2D CuS nanocrystals
HMNC	Hollow magnetite nanocluster
AuNCs@SiO_2_	Silica-encapsulated self-assembled gold nanochains
H-SiOx-PEG NPs	Hollow silicon oxide nanoparticles
CNPs	carbon materials
COFs	covalent organic frameworks
HPP	Hollow carbon nanosphere modified with polyethylene glycol-graft-polyethylenimine
QDs	Quantum dots
GQDs	Graphene quantum dots
EPR	Enhanced permeability and retention
CQDs	Carbon quantum dots
CCS	Cet-CDs-SNO
OPTAs	Organic photothermal agents
IPTAs	Inorganic photothermal agents
PPTAs	polymer-based photothermal agents
MPTAs	molecule photothermal agents
D-A	donor-acceptor
PBTPBF-BT	Thieno-isoindigo derivative-based D-A polymer
DPP-IID-FA	Diketopyrrole polymer
PDCDT	Semiconducting copolymer poly [(diketopyrrolopyrrole-cyclopentadithiophene)-ran-(diketopyrrolopyrrole-thiadiazoquinoline)]
ICG	Indocyanine green
FDA	Food and Drug Administration
PPy	Polypyrrole
Se	Selenium
Te	Tellurium
PDT	Photodynamic therapy
O_2_	Molecular oxygen
PS	Photosensitizer
ROS	Reactive oxygen species
CN-NPs	Carbon nitride nanoparticles
PTCDA	Perylene-3,4,9,10-tetracarboxylic dianhydride
SnO_2−x_@SiO_2_-HA	Oxygen-deficient black tin oxide nanoparticles
HA	Hyaluronic acid
ICB	Immune checkpoint blockade
CAR-T	Chimeric antigen receptor T-cell
TAAs	Tumor-associated antigens
DCs	Dendritic cells
APC	Antigen-presenting cell
bmNPs	biomimetic nanoplatforms
DAMPs	Damage-associated molecular patterns
ICD	Immunogenic cell death
GSH	Glutathione
CBI	checkpoint blockade immunotherapy
NK	Natural killer
CTLs	Cytotoxic T lymphocytes
ITME	Immunosuppressive tumor microenvironment
TAMs	Tumor-associated macrophages
CRT	Calreticulin
HMGB1	High-mobility group box 1 protein
MDR	Multidrug resistance
DOX	Doxorubicin
PCM	Phase change materials
H_2_O_2_	Hydrogen peroxide
AuMC	AuHNR@MnO_2_@CS
CS	Chitosan
TME	Tumor microenvironment
CDT	Chemodynamic therapy
BCC	Basal cell carcinoma
SCC	Squamous cell carcinoma
ISPI	In situ photoimmunotherapy
CLR	Complete local response
GSN	Gold silica nanoshells
LITT	laser interstitial thermal therapy
SCLC	Small cell lung cancer
NSCLC	Non-small cell lung cancer
LUAD	Lung adenocarcinoma
